# Effects of Digital Food Labels on Healthy Food Choices in Online Grocery Shopping

**DOI:** 10.3390/nu14102044

**Published:** 2022-05-13

**Authors:** Klaus L. Fuchs, Jie Lian, Leonard Michels, Simon Mayer, Enrico Toniato, Verena Tiefenbeck

**Affiliations:** 1 ETH AI Center, ETH Zurich, 8092 Zurich, Switzerland; etoniato@student.ethz.ch; 2 Institute of Computer Science (ICS-HSG), University of St. Gallen, 9000 St. Gallen, Switzerland; jie.lian@unisg.ch (J.L.); simon.mayer@unisg.ch (S.M.); 3 Institute of Information Systems, Friedrich-Alexander-Universität Erlangen-Nürnberg, 91054 Erlangen, Germany; leonard.michels@fau.de (L.M.); verena.tiefenbeck@fau.de (V.T.)

**Keywords:** Nutri-Score, digital food labels, food choice, randomized controlled trial (RCT)

## Abstract

In order to induce the shift in consumer behavior necessary for the mitigation of diet-related diseases, front-of-package labels (FoPL) such as the Nutri-Score that support consumers in their efforts to identify nutritionally valuable products during grocery shopping have been found to be effective; however, they remain non-compulsory in most regions. Counter-intuitively, a similar stream of research on digital web-based FoPL does not yet exist, even though such digital labels hold several advantages over physical labels. Digital FoPL can provide scalable and personalized interventions, are easier to implement than physical labels, and are especially timely due to the recent increase in online grocery shopping. The goal of this study was to demonstrate the technical feasibility and intervention potential of novel, scalable, and passively triggered health behavior interventions distributed via easy-to-install web browser extensions designed to support healthy food choices via the inclusion of digital FoPL in online supermarkets. To that end, we developed a Chrome web browser extension for a real online supermarket and evaluated the effect of this digital food label intervention (i.e., display of the Nutri-Score next to visible products) on the nutritional quality of individuals’ weekly grocery shopping in a randomized controlled laboratory trial (*N* = 135). Compared to the control group, individuals exposed to the intervention chose products with a higher nutritional quality (e.g., 8% higher healthy trolley index (HETI), 3.3% less sugar, 7.5% less saturated fat). In particular, users with low food literacy seemed to benefit from the digital FoPL (e.g., 11% higher HETI, 10.5% less sugar, 5.5% less saturated fat). Furthermore, participants exposed to the food label advocated its introduction more strongly than the control group (*p* = 0.081). Consumers worldwide could easily install such applications to display digital food labels on their end devices, and would thus not have to wait for stakeholders in the food industry to eventually reach consensus on mandatory food label introduction.

## 1. Introduction

The increase in diet-related diseases [1] has reached a global extent and become a major challenge to health-care systems around the world [2,3]. Although self-reported ambition to pursue healthy diets is almost omnipresent among consumers [4,5], the behavior of most individuals is driven by factors such as prices, tastiness, and practical considerations, and remains largely unchanged [6,7,8,9]. Prior research suggests that this gap between consumers’ intentions and their actual behavior is at least partially attributable to individuals’ use of simple non-compensatory heuristic strategies that rely on a few easily accessible pieces of information when making food-related decisions (e.g., [10,11]). Thus, health information is often overlooked due to its low salience and the effort that is commonly required to understand it [12].

In order to support healthy food choices, front-of-package labels (FoPLs) have been widely introduced [13,14]. Examples of such FoPLs include the French Nutri-Score (NS) and the Australian Health Star Rating [15]. In contrast to back-of-package declarations, which are usually in smal print [12,15,16], FoPLs present nutritional information in directly visible, condensed, and easy-to-interpret forms, thereby increasing the salience of health-related information and leading to improved nutritional quality of food purchases [14,15,17]. Because of their effectiveness, it seems counter-intuitive that in most regions of the world the provision of FoPLs is voluntary. As political decisions in the realm of food policy are typically controversial [18,19], only a few countries have managed to introduce FoPLs on a large scale, e.g., France [15,20,21]. In fact, there exists an ongoing urgent call by European scientists towards political stakeholders in the European Union to support the adoption of Nutri-Score in light of the scientific evidence regarding its effectiveness [20,21]. This delay between the availability of effective labelling solutions and their actual implementation could be bridged by empowering consumers themselves to employ labels in their food choice environments.

One such approach is to leverage digital technology in order to display food labels on user devices (e.g., laptops, smartphones) rather than requiring physical changes on product packages. In fact, a growing number of consumers are using their devices to order an increasing share of their of groceries online [22,23], a trend that has accelerated due to the circumstances surrounding the global COVID-19 pandemic [24]. In parallel, recent legislation by the European Union [25] on the mandatory declaration of nutrients for groceries sold online requires online grocery retailers to display the nutritional composition of products on their consumer-facing websites. This mandate has led to growing availability of structured product data via private and public food composition databases (FCDB), such as 1WorldSync, Atrify, and Open Food Facts.

These data can in turn be used to develop novel tools displaying FoPLs on online grocery websites, thus supporting users’ decision-making and potentially leading to beneficial behavior changes without requiring physical changes to product packaging [12,26,27,28,29].

Although digital food labels yield promising potential for researchers and consumers alike, there does not yet exist any published research or any empirical validation that involves web-based implementations of food labels for established retailers that run on end-user devices. The literature review that preceded this study revealed published research that leveraged digital tools mostly in computer-mediated framed field experiments on stationary laboratory computers usually programmed in the form of special-purpose software for the validation of food labels only [12,26,27,28,29,30,31]. Unfortunately, these framed experiments neither resemble realistic online grocery supermarkets nor stationary supermarkets, and therefore are unlikely to accurately reflect actual consumer behavior. In addition, the interventions of such experiments are not available for consumers outside of the laboratory setup, and therefore cannot support consumers in making healthier choices in the long term.

Hence, we set forth to develop and implement a web-based browser extension for consumers in Switzerland and Germany in order to validate the effects of a digitally displayed Nutri-Score on complete shopping baskets purchased by end consumers on a real online grocery shopping website (i.e., www.migros.ch, accessed on 1 February 2020). To the best of our knowledge, this study represents the first validation of a web-based system to display digital food labels in regular retailers’ online stores.

## 2. Materials and Methods

### 2.1. Research Questions

The present study investigated the following research questions (RQ). First, the immediate effect of the Nutri-Score (NS) food label on the dietary quality of food choices was assessed (RQ1). Because individuals with low food literacy are less receptive to contemporary nutrition-related awareness campaigns, RQ2 aimed to address the potential of digital food labels as an effective instrument among this group, as suggested by recent studies that proved this effect for printed food labels [32,33,34]. In addition, selective attention towards product attributes and information provided is considered as pivotal in determining the impact of a piece of information on subsequent decisions (e.g., [35]). Therefore, this study further investigated RQ3 on the role of actively perceiving digital food labels. Finally, via RQ4, any potentially detrimental affects of digitally labeling food products (such as greater reluctance among consumers [36,37]) were assessed.
**RQ1:***Does displaying product-specific Nutri-Score labels during the shopping process in an e-commerce environment lead to healthier immediate shopping behavior?***RQ2:***Does displaying product-specific Nutri-Score labels during the shopping process lead to healthier immediate shopping behavior among individuals with low food literacy?***RQ3:***Does displaying product-specific Nutri-Score labels during the shopping process have a particularly large impact on individuals who consciously perceived the label?***RQ4:***Does displaying product-specific Nutri-Score labels during the shopping process generate negative emotions and resistance towards their introduction?*

### 2.2. Dependent Variables

In order to assess whether digital labeling of food products with an NS leads to healthier immediate product choices, multiple dependent variables were analyzed. As there is no current scholarly consensus on the best metric(s) for assessing the nutritional quality of food product choices [38,39], the relative proportions of healthy food products (i.e., products labeled with an NS of A or B) and unhealthy food items (i.e., products labeled with an NS of D or E) were assessed. As the Nutri-Score framework is based on the established British Food Standards Agency nutrient profiling system (FSA-NPS DI), this categorization follows established principles. More concretely, for each study participant, the respective shares of healthy and unhealthy products within their shopping basket were compared based on weight share. In addition, the average NS of the shopping basket was adjusted for weight and the Healthy Trolley Index (HETI; [40]) (weighted in grams rather than Swiss Francs or Australian Dollars as in the original paper) of the shopping baskets was compared between the experimental and control groups. We chose to opt for a weight-based average of the Nutri-Scores within a basket for two reasons. First, to account for the European context of the study (i.e., where Nutri-Score is currently being adopted). Second, the weight-based average leads to more accurate results when products are selected that need to be processed prior to consumption. For example, it is unknown whether a person will decide to mix purchased cocoa powder with skim milk or whole milk at a later point in time. Hence, we decided to opt for weight-based averages, as the declaration of each product’s weight was available on the retailer’s website. Furthermore, we evaluated which elements of product information the participants had actually perceived during the shopping process and elicited their opinion towards the online supermarket based on the retailer trust scale developed by [41], which we extended with two additional items: “The online supermarket offers a healthy assortment” and “The online supermarket is transparent”. Moreover, we assessed to what extent participants approved the introduction of the NS label by different agents (i.e., manufacturers, nutrition experts, retailers, politicians). The above-mentioned scales were implemented using 5- or 7-point Likert scales. In the treatment group, we further elicited the participants’ emotional reaction to the displayed NS using a 7-point Kunin scale [42]; in both groups, we collected the perceived or anticipated intrusiveness and trustworthiness of the NS label using 7-point Likert scales. Finally, the participants’ food literacy was assessed using the Short Food Literacy Questionnaire (SFLQ) developed by Krause et al. [43] and previously validated in a German-speaking sample.

### 2.3. Instrumentation

In order to prepare for the assessment of the impact of digital food labels on actual food choices, we designed and implemented an information system in the form of an easy-to-install Chrome Web browser extension [44] that can mediate shoppers’ interactions with the website of a popular Swiss grocery chain (i.e., www.migros.ch, accessed on 1 February 2020).

**Figure 1:**
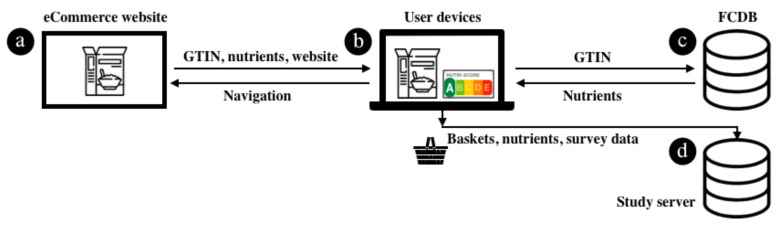
Nutri-Score information system (IS): Architecture and information flow. (**a**) e-Commerce system, (**b**) User devices, (**c**) public food composition databases (FCDB), (**d**) study server.

During the shopping process, the extension enriches product pages and product overview pages from the retailer’s e-Commerce system (at Figure 1a) with each product’s respective NS label (at Figure 1b). This is achieved by obtaining a product’s Global Trade Item Numbers (GTINs) and resolving them to nutritional information (at Figure 1c). To conduct the randomized controlled trial (RCT), we further adjusted the grocery chain’s website by removing promotions, customer ratings, guideline daily amount labels, and links to other websites. Beyond these adjustments, the Web browser extension did not affect individuals’ shopping experience. To support data assessment, the implemented system contained mechanisms to create user IDs, assign users to control and treatment groups, track users’ shopping process, and display introductory and post-study information and questionnaires. The collected data from interactions with users is provided (at Figure 1d).

### 2.4. Statistical Assessment

The generated shopping and survey data from the randomized controlled trial were assessed using statistical t-tests in order to determine whether any significant differences existed between the means of the two groups, i.e., the treatment group that received the digital NS food labels and the control group that did not. More specifically, we used t-tests after confirming that the sample data were normally distributed using a Shapiro-Wilk test to compare the treatment and control groups regarding the nutritional quality of their food choices in respect of the self-reported survey data. Prior research has shown that significant differences between groups are generally difficult to obtain for RCTs in the context of food and nutrition [45,46,47] due to the typically large variance in food and nutrition data. As our experiment took place in a controlled laboratory environment, we are confident that our RCT approach is suitable for obtaining meaningful results; however, in combination with our limited sample size, the high variance in food and nutrition data might overshadow true effects. Therefore, we used a significance level α of 10% as threshold in evaluating differences between the two groups. As we did not find any significant differences in terms of effects between the Swiss and German samples, we merged the data of the two samples and do not differentiate between the two countries.

### 2.5. User Study

The user study was conducted in two on-campus computer-equipped behavioral laboratories at Universities in Switzerland and Germany in Q1/2020 (i.e., before the beginning of the COVID-19-related lockdowns and closure of university campuses). As this was our first study in the field of leveraging a Chrome extension for diet-related behavior change interventions, we opted for a realistic laboratory-based study setup in order to control for moderating effects, and refrained from conducting a study in the public domain due to several related challenges. First, website changes by retailers are frequent and can require design changes in the website parsing function of the extension via timely over-the-air-updates to the users’ end devices. Second, to avoid moderating effects of promotions, the manuscript authors removed all advertisements and all links to external websites via the Chrome extension. Logically, such an extension would not be of popular interest in the public Chrome extension store as it limits the usability of eCommerce websites. Lastly, this study was intended to demonstrate the overall feasibility of such user-mediated digital food labels and to serve as a preliminary study on the path to larger public-field studies in the future, not to create a consumer product intended for long-term usage. In both labs, our Nutri-Score browser extension was pre-installed on all workstations. We recruited 135 participants, who received financial compensation of CHF 20 or EUR 10 (depending on lab location) as an incentive for completing the shopping task and subsequent questionnaire, which required one hour. To achieve incentive compatibility and induce participants’ truth-telling regarding their actual product preferences, they entered a lottery to win the products they chose during the task. After obtaining consent and completing a demographic questionnaire, we asked participants to carry out their weekly grocery shopping in an online supermarket with a predefined fixed budget (EUR 55 in Germany and CHF 100 in Switzerland, with values adjusted for differences in purchasing power). In order to minimize misunderstandings, the task description was read aloud before the study began and could be accessed during the shopping phase. Next, participants received instructions on how to use their virtual shopping cart to add and remove products as well as about the pieces of information displayed in the detailed view of a product. For the treatment group, the Nutri-Score system was briefly and unobtrusively described among other elements by showing a screenshot of a detailed product page including descriptions for every element on that page (e.g., product price or a picture of the product).

Participants then began to shop in the online store of a large Swiss retail company. The assortment of food products was not restricted, and contained well over ten thousand products. For the German sample, the online supermarket showed prices converted to Euros. The conversion factor comprised the currency exchange rate and a product category-specific factor adjusting for the different price levels in the two countries. There was no time limit set for the shopping task. After participants had submitted their shopping basket, they were redirected to a post-task online questionnaire that assessed specific dietary restrictions of the participants and multiple aspects regarding trust toward the retailer, self-reported food literacy, and the participant’s approval of the Nutri-Score system.

**Figure 2:**
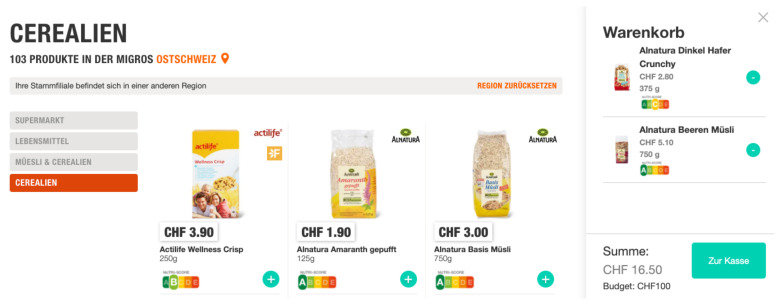
User study: Treatment group view of a category on the online grocery shopping website.

**Figure 3:**
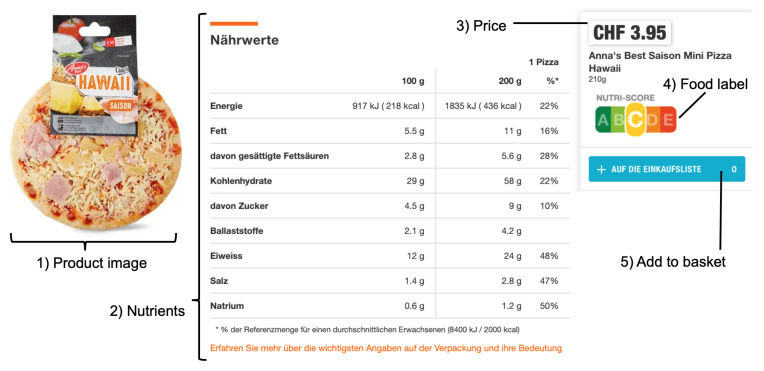
User study: Information available for treatment group in the specific product view on the online grocery shopping website (information composed and descriptions added).

**Figure 4:**
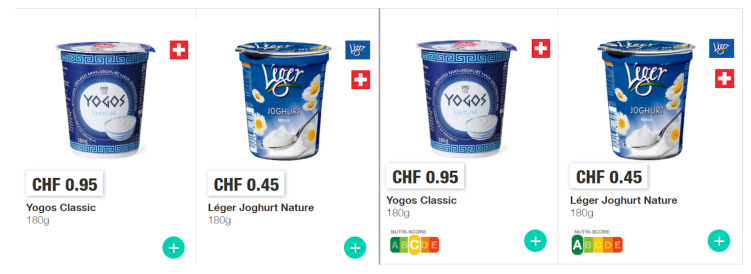
Comparison of two products as seen by the control group (**on the left**) and treatment group (**on the right**).

To evaluate the impact of the NS label on participants’ product choices and shopping experience, we manipulated whether products in the online supermarket were labeled with their corresponding NS or not. In the treatment group (TG), NS labels were visible on all products, as would be the case for a mandatory introduction of NS labels. More precisely, NS labels were displayed in the treatment group both in the category overviews (see Figure 2) and in the individual product views (see Figure 3). By contrast, no NS labels were displayed in the Control Group (CG) (see Figure 4).

Participants were randomly assigned to one or the other experimental condition, aiming for a similar distribution of gender, age, food literacy, and income level across the treatment and control groups. Based on prior research, we expected participants in the labeling condition to choose healthier food products than participants in the control condition. Furthermore, we hypothesized that participants with low food literacy in the treatment group would choose healthier food products than those with low food literacy in the control group. Finally, our last hypothesis was that those users in the treatment group who consciously perceived the NS label during their shopping would choose particular more healthy food products than participants in the control group. The study protocol was approved by the ethics commission of ETH Zurich (approval granted by the ethics commission of ETH Zurich on 17 December 2019, ethics approval request 2019-N-177: eCommerce Widget for Nutrition and Sustainability).

### 2.6. Participants

We recruited 135 participants, who took part in the study in one of twelve sessions. Our sample consisted wholly of university students, who were invited to participate in the study through emails sent from the behavioral laboratories to their databases of registered interested students. The behavior laboratories at both locations offer professional study setups and guarantee via ID check that no participant can participate multiple times at either location. In addition, the theoretical chance that a participant could have joined at both study locations can be excluded, as both labs require a student to be registered at the local university. Following plausability checks, nine participants’ data had to be excluded due to incomplete data, extremely short duration of the experiment, and technical difficulties; thus, the final sample consisted of 126 participants. Table 1 provides an overview of the participants’ demographic data.

## 3. Results

### 3.1. Randomization Check

The treatment group and the control group did not differ significantly regarding gender distribution ($\chi^{2}$(1) = 1.07, *p* = 0.301), age (*t*(124) = 0.13, *p* = 0.895), education level (*p* = 0.700), or income level (*p* = 1). There were no differences regarding the distribution of specific diets across the two groups except for a high-protein diet that was more prevalent in the treatment group compared to the control group (*n* = 22 vs. *n* = 12; $\chi^{2}$(1) = 5.04, *p* = 0.025).

### 3.2. Manipulation Check

In order to check whether participants perceived the NS labels, and thus our experimental manipulation, the post-task questionnaire asked participants to state which out of ten product information elements they had perceived during the shopping task, resembling an aided recall task. Of the TG participants, 71.2% recalled having seen the NS label. In the control group, where none of the participants had in fact been exposed to the NS label, a single participant falsely affirmed having seen that element.

### 3.3. Nutritional Quality of Food Choices

Our analysis of the participants’ shopping baskets, summarized in Table 2, indicates that displaying the NS label on food products during the shopping process led to healthier shopping behaviors. The mean HETI in the treatment group is significantly higher than in the control group (*p* = 0.068). For the three other indicators, namely, average NS and the proportions of healthy and unhealthy food products, the results point in the same direction; however, they did not reach statistical significance. We further analyzed whether the two groups differed regarding the mean nutritional characteristics of the purchased food items per 100 g. The results are summarized in Table 3. For saturated fat, sugar, and unhealthy sugar, the treatment group showed a significant reduction compared to the control group, which was associated with healthier food product choices. For all other nutrients the treatment effects were not significant, though they point in the same direction. Overall, these results are in line with our first hypothesis.

In order to assess the effect of our Nutri-Score instrumentation on individuals with low food literacy, we compared the shopping baskets of participants whose subjective food literacy score was in the lowest third (first tertile) in the treatment and the control group. The general results in Table 2 do not indicate a significant effect of the Nutri-Score IS on the food choices among that subgroup. However, a more detailed analysis of the purchased products’ nutritional characteristics in Table 3 reveals that the mean amount of sugar and unhealthy sugar was significantly lower in the TG shopping baskets compared to those of the CG. For all other nutrients the treatment effect was not significant, although pointing in the same direction. These results are generally in line with our second hypothesis, though supporting it only on a descriptive level.

With respect to our third hypothesis, we tested the effects of Nutri-Score IS on the nutritional quality of product choices for participants in the TG who stated that they had perceived the NS label during the shopping process. Due to the non-invasive design of the empirical study, *N* = 17 users (ca. 29%) responded that they did not perceive the NS label during the shopping task. Surprisingly, one user within the control group responded that he had seen the NS label, which was technically not possible. Hence, he was excluded from this analysis. When comparing members of the TG, users who reported perceiving the NS labels (TG${}_{perceived}$: *N* = 42) purchased significantly healthier foodstuffs by weight-averaged Nutri-Score (*p* = 0.035) than users in the TG who reported not perceiving the label (TG${}_{not-perceived}$: *N* = 17). Compared to participants in the CG, these participants chose a significantly higher proportion of healthy food, a significantly lower proportion of unhealthy food, and had a marginally significantly higher HETI. The difference in the average NS was not significant, though it pointed in the same direction. A more detailed analysis of the purchased products’ nutritional characteristics is provided in Table 3, and shows that participants who stated that they saw the NS label bought products with a significantly lower amounts of saturated fat compared to the CG. For all other nutrients there was no significant difference between groups. These results are in line with our third hypothesis.

### 3.4. Effects on User Perception

In order to gain a deeper understanding of the effects of displaying NS labels on participants’ overall response to the Nutri-Score and the online supermarket, we analyzed whether the TG and the CG differed regarding the dependent variables assessed in the post-experimental questionnaire. We did not find significant differences between the two groups in terms of their rating of the overall shopping experience (*t*(124) = −0.24, *p* = 0.813). Regarding the retailer trust scale, adapted from [41] and extended by two items (overall Cronbach’s α = 0.72), the two groups did not differ significantly (*t*(124) = −0.31, *p* = 0.758). No significant differences between groups were found in their assessments of the NS labels as intrusive (*t*(124) = −0.40, *p* = 0.687) or trustworthy (*t*(124) = −0.14, *p* = 0.890).

In order to investigate our fourth research question, we analyzed the treatment group’s responses on a 7-point Kunin scale assessing their emotional response to the displayed NS labels. This analysis reveals that the emotional response was slightly positive (*M* = 4.90, *SD* = 1.00) and differed significantly from the neutral response of 4 (*t*(58) = 6.94, *p* < 0.001). Regarding the implementation of Nutri-Score labels by food producers, support in the TG (*M* = 4.86, *SD* = 1.69) was marginally higher (*t*(124) = −1.82, *p* = 0.071) compared to the CG (*M* = 4.28, *SD* = 1.87). Concerning potential NS introduction by other groups of stakeholders, the differences were non-significant (politicians: *t*(124) = −1.01, *p* = 0.314; independent nutrition experts: *t*(124) = 0.04, *p* = 0.967; retailers: *t*(124) = −1.46, *p* = 0.147. Averaging the approval scores of the multiple agents to a general approval score (CG: *M* = 4.41, *SD* = 1.70; TG: *M* = 4.92, *SD* = 1.55) revealed marginally significantly higher general approval of a Nutri-Score implementation by the TG (*t*(124) = −1.76, *p* = 0.081) compared to the CG. These results suggest that displaying NS labels via Nutri-Score IS does not lead to negative emotional responses or resistance towards the introduction of such a system.

## 4. Discussion

In this study, we proposed a Web-based digital tool in the form of a browser extension that can be installed on end users’ client devices to automatically display digital food labels in the form of Nutri-Scores for food items viewed during online grocery shopping. NS labelling can support consumers in making healthy food choices. In order to assess how this extension affects actual grocery shopping behavior, we conducted a user study with 135 participants. We divided our participants into control (CG) and treatment (TG) groups and proposed a set of dependent variables, including the proportion of healthy food products, the proportion of unhealthy food products, the average Nutri-Score of the shopping basket, and the Healthy Trolley Index (HETI), to compare the participants’ shopping decisions in terms of nutritional quality.

With regard to our first research question, the resulting evidence indicated that using the web-based extension to display food product Nutri-Scores in the online supermarket led to healthier food product choices. However, we only found significant results with regard to the HETI as an indicator of purchase nutritional quality. For the average Nutri-Score and proportion of healthy and unhealthy food products, we found only descriptive effects suggesting a positive effect of our intervention. In a more detailed analysis of the nutrient contents of the participants’ purchases, we found significant positive effects of the Nutri-Score on the average saturated fat, sugar and unhealthy sugar contents of the selected products. These results partly support the findings of prior research regarding the effects of food labels in online supermarkets. Unlike Finkelstein et al. [26], we did not find a significant effect of our intervention adding Nutri-Scores to a real-life online grocery shopping website or on the average Nutri-Score of the purchased products. However, we did find a positive effect on specific nutrients, which their experiment did not reveal. The key differences between the study of Finkelstein et al. [26] and the one reported in this manuscript is that our study used a between-subject design to investigate the effects of displaying Nutri-Scores in an online-supermarkets. Thus, our participants only interacted with the supermarket once in the course of the experiment. In addition, we used an existing, large online supermarket for our experiment instead of the artificial one constructed by Finkelstein et al. [26]. Lastly, due to our between-subject design and limited sample size our study suffered from a lack of statistical power, which might overshadow additional effects of our intervention. Other studies in the domain of FoPLs in online supermarkets included much larger samples [27,28,29], specifically asked participants to shop for healthy products [28], or used introductory videos to explain the food labels, which might have primed the participants [26,29]. These differences might explain why other studies have reported larger effects for FoPLs in online supermarkets. In general, the relatively small effects reported in our study are in line with other studies assessing the effects of FoPLs on the nutritional quality of food choices (e.g., [48,49,50]).

Our second research question involved assessment of the impact of digital food labels on the shopping behavior of consumers with low food literacy, which is an at-risk population for diet-related diseases. Low food literacy users in the treatment group selected food items that featured significantly lower amounts of sugar and unhealthy sugar (Table 3). In addition, though it did not reach statistical significance, they made healthier food choices with regard to all other measures, such as HETI and the share of healthy and unhealthy food items. On average, they made healthier choices in terms of saturated fat, protein, dietary fiber, and sodium. In conclusion, the study indicates that low food literacy consumers can potentially benefit from digital food labels.

With regard to our third research question, we assessed the effect of the conscious perception of NS labels. In theory, the conscious perception of a digital food label should amplify the effects of such an intervention due to the increase in salience and the reduction in search costs. We conclude that the displaying of digital food labels has significant beneficial impacts on the healthy food choices of individuals who consciously perceived the NS label. The other nutrients and indicators (HETI, share of healthy and unhealthy products) suggest that both groups, i.e., TG${}_{perceived}$ and TG${}_{not-perceived}$, made similarly healthy purchase decisions. More research is needed to understand the impact among members in the TG better, as users who did not report perceiving the NS label made healthy shopping decisions as well. Hence, conscious perception of the label does not seem to correlate with negative effects. As the Nutri-Score is a relatively new concept, users might not be fully aware of its purpose, which may explain their not consciously noticing the digital label. We suggest that users of such an intervention should receive a salient introductory tutorial on the purpose of the digital NS label to ensure that it is well understood.

For our final research question, we assessed participants’ perception of the Nutri-Score and of their shopping experience. Members of our control and treatment groups neither differed in their rating of the overall online shopping experience nor in their rating of the Nutri-Score’s intrusiveness or trustworthiness. The digital food labels were perceived as slightly positive by the TG, with their rating being significantly different from neutral. Hence, we conclude that the introduction of such web-based information systems (for instance by food producers, as suggested by the TG members in this study) can be expected to not lead to negative emotional reactions or resistance. When asked about future mandatory introduction of the NS label, users in the TG were more favorable to its introduction than their counterparts in the CG. This result is in line with the findings of Cadario et al. [50], who found that cognitive nudges such as evaluative food labels are generally accepted by consumers. Hence, in order to facilitate the ongoing political debate and process of Nutri-Score introduction (e.g., current the Nutri-Score petition in Europe), IS tools such as the proposed browser extension can help both citizens and political decision-makers to understand the potential of these labels prior to introduction.

### 4.1. Contributions

This study contributes to research on FoPL and their web-based realization in the form of IS. First, our empirical field study confirms the beneficial health impact of web-based FoPLs on food choices. More specifically, our findings suggest that members of the TG who were exposed to IS-mediated digital food labels made healthier purchasing decisions, indicating that NS labels increase users’ attention to the nutritional quality of food when selecting products. Second, our findings show that Web-based IS options which enrich online grocery stores with NS labels are a promising approach to improving shoppers’ food choices. This is especially promising as such an IS does not rely on active user input, and can automatically display relevant just-in-time interventions during the online grocery shopping journey. In addition to its contribution to the literature, this study has important practical implications. Providers of online grocery websites and food producers need to understand that such an IS can be installed by consumers themselves on their own devices with little possibility of intervention; as healthy food choices are a goal that most consumers aspire to follow, there exists the possibility of mass adoption of such IS applications in the future even if distributors and producers do not agree. Therefore, such IS options could induce a shift in consumer behavior towards healthier food choices with consequential negative commercial implications for producers of primarily unhealthy food items. This includes the potential adoption of NS labels by consumers in regions where food labels such as Nutri-Score have not yet been mandated by regulators due to resistance from industry or regulators. Furthermore, health-care professionals such as physicians and dietitians could recommend that their patients use such an IS. As the Nutri-Score IS is triggered automatically while making purchasing decisions during online grocery shopping, the system does not require active user input such as permanent logging of dietary information. As manual diet-logging applications are often discontinued by a majority of users, automatic IS-based interventions such as our Nutri-Score IS offer a promising complementary approach to current mobile dietary health applications.

Finally, this study has the potential to impact regulatory decision-making on mandating machine-readable declarations of nutrients on food products sold online in the future. Today, despite the existence of regulatory mandates for food declaration with respect to groceries sold online (e.g., EU1169/2014), there does not yet exist a machine-readable standardized format for use across a majority of e-Commerce retailers. Hence, developing web-based applications that assist consumers in making healthier food choices in online grocery shopping, such as the one developed for this study, should be adopted for every retailer and updated whenever the retailer changes its website setup. Consumers stand to benefit if online grocery retailers adopt a standardized syntax (e.g., linked data, GTIN on the Web by GS1) which would allow Web-based applications (e.g., Chrome extensions) to parse websites from multiple retailers without requiring significant adaptations for each retailer’s website. If such a standard existed, search engines and shopping assistants would be able to parse products’ nutritional data, including allergens, nutritional composition, labels, and ingredients across retailers, at far lower costs and with potentially even higher efficacy (as products could be compared across retailers) thanks to the reduced setup, thereby allowing more consumers to benefit from such web-based tools in making healthier food choices online.

### 4.2. Limitations

Certain limitations of this empirical field study exist. First, the number of participants was limited, as the study design followed an in-person setup for which participant recruitment had to be terminated due to the ongoing COVID-19 pandemic in Q2/2020. The number of participants ought to be increased in future studies in order to further confirm the promising potential of digital food labels in online grocery shopping. In addition, as the participants were university students their shopping behavior and reaction to the digital food labels might not be representative of the entire population. The study was performed in a laboratory setting with clear shopping guides as well as environmental controls and while excluding incentives such as discounts. Therefore, it is difficult to precisely simulate participants’ daily shopping behaviour in the real world, where such distractions might moderate the impact of digital food labels. In addition, the extension was designed only to track participants’ shopping behavior during a single session, while improvements in dietary behavior usually require long-term interventions and monitoring of food choices. Such long-term shopping assessments across multiple purchasing sessions could be supported in a future version of the browser-based extension. A data-minimal approach was selected in regard to data collection in order to prevent re-identification of individual users, as suggested by our universities’ ethics commissions; hence, the questionnaire that was submitted for ethical approval did not collect information on the students’ faculty affiliation. Potential stratification of the user sample across different study majors could potentially reveal the potentially moderating role of preexisting dietary knowledge in the context of digital label efficacy. As neither university involved in this study had any programs in place focusing specifically on food science or related disciplines, the study population needs to be extended in future follow-up research in order to further evaluate the potential of digital food labels to support consumers with differing levels of food literacy. Furthermore, the classification of healthy and unhealthy food items based on the Nutri-Score framework is debatable. As there is no scholarly consensus on metrics for assessing the nutritional quality of food product choices [38,39], investigators assessing such mechanisms to measure behavioral change need to carefully select their dependent variables. In fact, it can be argued that such data from food packs (including labels as well as health and nutritional claims) might by themselves be insufficient to consider one product healthier than others [38,39]. Similarly, the Nutri-Score framework serves as an imperfect approximation of dietary quality, and is not a substitute for professional dietary counseling by health-care providers or dietitians. For example, the label does not account for important factors that influence current individual dietary needs, such as body composition, recent dietary and physical activity, food allergies, or the diet-related diseases of a specific user. In addition, comparison on the basis of 100 g amounts is a non-ideal unit of measurement for diet counseling. In fact, portion sizes would probably represent a more realistic approximation of the amount that a user will ultimately consume from a specific product at a later stage in time after the purchase. Thus, there exists the potential for future studies to design and validate the potential of novel digital food labels that leverage information about the individual user in order to deliver improved tailored interventions. Lastly, while the average protein values in the treatment group were higher compared to the control group, the post-experimental questionnaire shows a higher prevalence of daily protein intake in the treatment group compared with the control group, and it is unclear whether this increase in protein intake was caused by the display of the Nutri-Score labels.

## 5. Conclusions

Providing an interpretive FoPL (i.e., the Nutri-Score) in an established online supermarket can increase the nutritional quality of food products chosen by consumers according to the HETI. This result provides additional support for the wide-spread implementation of such labels. This is especially emphasized as we found no difference in effects between participants with low and high levels of food literacy. Finally, we found that providing Nutri-Score labels in a real online supermarket led to positive consumer perceptions and higher general approval ratings regarding its implementation.

## Figures and Tables

**Table 1:** Demographic summary of participants.

Sample	Total (*N* = 126)		Switzerland (*N* = 53)		Germany (*N* = 73)	
	CG (*N* = 67)	TG (*N* = 59)	CG_CH_ (*N* = 29)	TG_CH_ (*N* = 24)	CG_DE_ (*N* = 38)	TG_DE_ (*N * = 35)
Age [yrs]	23.42 ± 3.31	23.34 ± 3.41	23.24 ± 3.45	22.63 ± 2.69	23.55 ± 3.23	23.83 ± 3.86
Female (%)	33 (49.25)	36 (61.02)	10 (34.48)	10 (41.47)	23 (60.53)	26 (74.29)
Male (%)	34 (50.75)	23 (38.98)	19 (65.52)	14 (58.53)	15 (39.47)	9 (25.71)
Sec. school ^1^ (%)	40 (59.70)	33 (55.93)	17 (58.62)	12 (50.00)	23 (60.53)	21 (60.00)
Tert. school (%)	27 (40.30)	26 (44.07)	12 (41.38)	12 (50.00)	15 (39.47)	14 (40.00)
Income ^2^	1543 ± 1137	1687 ± 1303	1700 ± 1442	1738 ± 1444	1440 ± 876	1657 ± 1226
Food Literacy ^3^	35.29 ± 6.66	35.90 ± 7.12	33.54 ± 6.79	35.22 ± 8.53	36.66 ± 6.22	36.37 ± 5.93

Mean values with SD, relative frequencies in parentheses; ^1^ Secondary school completed; ^2^ total in Germany in EUR (1 EUR = 1.05 CHF); ^3^ SFLQ [43]; CH: Control group, TG: Treatment group; CH: Switzerland, DE: Germany.

**Table 2:** Results of shopping basket analysis for control group (CG) and treatment group (TG).

All Users	CG (*N* = 67)	TG (*N* = 59)	*p*
Purchased food quantity (g) ^1^	16,299 ± 6420	15,762 ± 6742	
Average Nutri-Score ^1,5^	3.67 ± 0.47	**3.75** ± 0.30	0.33
Healthy food ^2,3^	77.1 ± 12.9	**80.4** ± 12.1	0.15
Unhealthy food ^2,3^	12.5 ± 10.2	**10.5** ± 9.87	0.26
HETI ^4^	54.9 ± 13.1	**59.3** ± 13.4	0.068 *
**Low Food Literacy Users**	**CG_LFL_ (** ***N*** ** = 27)**	**TG_LFL_ (** ***N*** ** = 21)**	***p***
Purchased food quantity (g) ^1^	15,636 ± 6620	16,232 ± 5820	
Average Nutri-Score ^1,5^	3.67 ± 0.47	**3.75** ± 0.30	0.33
Healthy food ^2,3^	76.6 ± 12.6	**77.8** ± 13.7	0.75
Unhealthy food ^2,3^	14.4 ± 9.7	**12.7** ± 10.7	0.57
HETI ^4^	53.6 ± 13.5	**59.5** ± 15.8	0.19
**Conscious Perception**	**CG_not-perceived_ (** ***N*** ** = 66)**	**TG_perceived_ (** ***N*** ** = 42)**	***p***
Purchased food quantity (g) ^1^	14,325 ± 6542	16,326 ± 4765	
Average Nutri-Score ^1,5^	3.67 ± 0.47	**3.75** ± 0.30	0.33
Healthy food ^2,3^	77.1 ± 13.1	**81.5** ± 11.7	0.077 *
Unhealthy food ^2,3^	12.6 ± 10.3	**9.4** ± 9.0	0.094 *
HETI ^4^	54.9 ± 13.2	**59.1** ± 12.8	0.11

^1^ mean + SD; ^2^ % ± SD; * signiﬁcant at α = 0.1; ^3^ share of weight of (un-)healthy food items with Nutri-Score A or B (D or E) among all foodstuffs in total basket; ^4^ Healthy trolley index (HETI) by weight, food only (scale from 0 (unhealthy) to 100 (healthy)); ^5^ Nutri-Score averaged by product weights (scale 0.5 (E) to 4.5 (A)); **bold** marks healthier values in direct comparison of group G_1_ and G_2_ if |*n*_G_1__ − *n*_G_2__|/|*n*_G_1__| ≥ 2%; CG: Control group, TG: Treatment group, LFL: Low food literacy, g: grams.

**Table 3:** Detailed analysis of average nutrients per 100 g in shopping baskets in control group (CG) and treatment group (TG).

All Users	CG (*N* = 67)	TG (*N* = 59)	*p*
Energy ^2^	623.55 ± 91.94	637.38 ± 76.09	0.38
Saturated fat ^1^	1.87 ± 0.24	**1.80** ± 0.23	0.070 *
Sugar ^1^	6.16 ± 0.77	**6.02** ± 0.73	0.100 *
Unhealthy sugar ^1,3^	3.84 ± 0.79	**3.52** ± 0.71	0.100 *
Protein ^1^	5.14 ± 0.81	**5.49** ± 0.66	0.78
Dietary fiber ^1^	1.84 ± 0.26	**2.12** ± 0.26	0.47
Sodium ^1^	0.39 ± 0.25	**0.32** ± 0.12	0.34
**Low Food Literacy Users**	**CG_LFL_ (** ***N*** ** = 27)**	**TG_LFL_ (** ***N*** ** = 21)**	***p***
Energy ^2^	637.81 ± 131.63	623.21 ± 124.90	0.37
Saturated fat ^1^	2.09 ± 0.42	**1.98** ± 0.38	0.23
Sugar ^1^	7.05 ± 1.43	**6.31** ± 1.28	0.097 *
Unhealthy sugar ^1,3^	4.74 ± 1.51	**3.71** ± 1.24	0.072 *
Protein ^1^	5.17 ± 1.28	**5.19** ± 1.02	0.55
Dietary fiber ^1^	1.82 ± 0.38	**1.96** ± 0.40	0.96
Sodium ^1^	0.31 ± 0.14	**0.26** ± 0.17	0.41
**Conscious Perception**	**CG_not-perceived_ (** ***N*** ** = 66)**	**TG_perceived_ (** ***N*** ** = 42)**	***p***
Energy ^2^	622.30 ± 92.92	628.53 ± 92.30	0.59
Saturated fat ^1^	1.88 ± 0.24	**1.71** ± 0.26	0.055 *
Sugar ^1^	6.17 ± 0.77	**5.85** ± 0.86	0.16
Unhealthy sugar ^1,3^	3.86 ± 0.80	**3.44** ± 0.86	0.18
Protein ^1^	5.14 ± 0.82	**5.40** ± 0.80	0.95
Dietary fiber ^1^	1.83 ± 0.26	**2.11** ± 0.32	0.31
Sodium ^1^	0.39 ± 0.26	**0.29** ± 0.14	0.28

^1^ mean + SD (in g) per 100 g of purchased food; ^2^ mean + SD (in KJ) per 100 g of purchased food; ^3^ amount of sugar contained in purchased products (except fruits and vegetables). CG: Control group, TG: Treatment group, LFL: Low food literacy, * signiﬁcant at α = 0.1; bold marks healthier values v in direct comparison of group G_1_ and G_2_ if |*v*_G_1__ − *v*_G_2__|/|*v*_G_1__| ≥ 2%.

## Data Availability

The data presented in this study are available on request from the corresponding author. The data are not publicly available due to the nature of the ethics approval request and the participants’ consent to make their data available for research only.
